# Achieving consensus on the curriculum system for central sterile supply department nurses: a modified Delphi study

**DOI:** 10.3389/fmed.2026.1774004

**Published:** 2026-05-07

**Authors:** Jiawei Liu, Nian Qin, Fengliu Gui, Hui Chen

**Affiliations:** 1 Central Sterile Supply Department, West China Hospital of Sichuan University, Chengdu, China; 2 West China School of Nursing, Sichuan University, Chengdu, China

**Keywords:** central sterile supply department, competencies (outcomes), Delphi technique, nursing, training framework

## Abstract

**Background:**

Enhancing the core competencies of Central Sterile Supply Department (CSSD) nurses is of paramount importance, as these professionals serve as the frontline defense against healthcare-associated infections. Accordingly, this study aimed to develop an evidence-based training framework for CSSD specialists to provide a standardized benchmark for continuous professional development in the CSSD field.

**Methods:**

The initial development of the CSSD nurse training system involved an extensive literature review and expert group discussions. From April to June 2024, two rounds of Delphi surveys were conducted, consulting 25 experts in relevant fields from nine provinces across China to reach consensus on the index system. Descriptive statistics were employed for data analysis. Consensus thresholds were defined as a mean score of ≥3.5, a coefficient of variation of <0.25, and a full-score percentage of ≥70%.

**Results:**

The response rates for the two rounds of expert consultation were 92.59 and 100%, respectively, with an expert authority coefficient of 0.912. In the first round, 13 items were revised, 4 deleted, 2 repositioned, and 20 added; no changes were made in the second round. This resulted in a training process index system comprising 5 first-level, 12 s-level, and 50 third-level indicators, and a training content index system comprising 4 first-level, 24 s-level, and 107 third-level indicators. Kendall’s W coefficients for the training process and training content increased from 0.199 and 0.187 in the first round to 0.227 and 0.218 in the second round (*p* < 0.001), indicating improved expert consensus.

**Conclusion:**

This training index system is both scientifically sound and reliable, offering a valuable reference for specialist nurse selection, curriculum design, and quality assurance in CSSD training programs.

## 1 Introduction

The Central Sterile Supply Department (CSSD) is responsible for the cleaning, disinfection, sterilization, and distribution of all reusable medical instruments and supplies (1). However, professional development in this field has lagged behind other clinical specialties, and CSSD nursing education remains less structured than that that provided for bedside nurses (2). As both a high-concentration area for pathogenic microorganisms and the primary source of sterile supplies for the entire hospital, the CSSD represents a critical control point for infection prevention. Quality control failures in this setting can result in sterilization breaches, cross-contamination between clean and contaminated items, and ultimately, healthcare-associated infections that may endanger patient lives (3–5). With evolving infection control standards and increasingly stringent regulatory requirements, hospitals now place greater emphasis on nosocomial infection prevention. This places higher demands on the comprehensive competence of CSSD personnel, who are responsible for managing the reprocessing and distribution of sterile supplies. Consequently, strengthening the professional training and skill development of CSSD staff has become a pressing priority.

High-quality workforce development depends on robust training programs. Sterile processing is a highly specialized discipline spanning clinical care, microbiology, and the physical sciences (3). In China, CSSD staff are predominantly nurses; however, pre-service nursing curricula typically exclude sterile supply management (6), necessitating comprehensive on-the-job training to develop specialized expertise. National standards issued in 2009 mandate continuing education for CSSD personnel, with competency-based training and regular knowledge updates (7). Similarly, the 2012 edition of *Disinfection Technical Specifications* emphasizes enhanced training for sterilization personnel (8).

The Asia Pacific Society of Infection Control strongly recommends regular continuing education and recertification for sterile processing personnel in its healthcare facility guidelines. All staff engaged in medical device reprocessing must complete formally recognized sterilization technology training, receive ongoing supervision, undergo periodic competency assessments, and maintain function-specific proficiency through regular in-service education (9). Furthermore, both the World Health Organization and the Pan American Health Organization emphasize that personnel at all levels must demonstrate competence in their assigned roles, highlighting the need for comprehensive training programs. Continuous professional development—keeping CSSD staff abreast of advances in sterilization science, instrumentation, and technological innovations—is therefore essential (10).

In many developed countries, many CSSD staff have prior surgical or clinical experience. The WHO explicitly cautions against allowing untrained personnel to perform device reprocessing without proper certification. Certification requires periodic renewal through documented continuing education credits; failure to maintain certification results in credential suspension (10). The WHO outlines a tiered educational framework for CSSD personnel: entry-level operators require secondary education; intermediate technicians require 2 years of specialized training and basic CSSD curriculum completion; senior supervisors require 5 years of experience and intermediate curriculum completion; and managerial positions require eight or more years of experience with advanced curriculum completion (10).

In China, the Beijing PLA General Hospital pioneered CSSD specialist nurse training in 2013, followed by the Beijing Nursing Society’s launch of certification programs in 2014 (11). Subsequently, multiple provinces—including Shaanxi, Guangdong, Chongqing, Sichuan, Yunnan, Hunan, and Heilongjiang—implemented provincial-level training initiatives (12–14). In 2021, the Chinese Nursing Association established the first national training program for CSSD specialist nurses (15). While the Chinese Nursing Association has established a foundational national training framework for CSSD specialist nurses, operational guidance for localized implementation—particularly regarding trainee and instructor qualifications, process-based assessment, and comprehensive competency development—remains underdeveloped; this study addresses these specific gaps by developing a detailed, implementable index system that complements and extends the national standards.

Expert consensus methods are essential for developing valid training frameworks, as specialist input provides greater reliability than non-expert opinion. The Delphi technique is a widely recognized approach for systematically eliciting and aggregating expert judgments to reach consensus on complex issues (16). Grounded in iterative rounds of structured consultation, the Delphi method refines questionnaire items based on feedback from previous rounds, enabling progressive convergence of expert opinion (17). The modified Delphi variant streamlines this process by replacing the initial open-ended inquiry with evidence-based preparation—typically a literature review and expert panel input—thereby reducing the number of iterations required and accelerating consensus achievement (18, 19). Given its established utility in curriculum development and competency framework design, this study adopted the modified Delphi approach.

## 2 Methods

### 2.1 Design

A modified Delphi method was used to develop the training framework. This approach comprises two phases: (1) an initial preparatory phase involving a comprehensive literature review and expert panel discussions to establish the preliminary indicator pool; (2) a consensus phase employing iterative expert surveys to refine the index system until stability is achieved. The Delphi method ensures structured, anonymous expert input while preventing undue influence among panel members, thereby enhancing the reliability of consensus outcomes (20). Our research team comprised a project director, clinical specialists, and research assistants, with responsibilities that included literature synthesis, expert panel recruitment, instrument design and distribution, data collection, and statistical analysis.

#### 2.1.1 Phase 1: drafting the expert consultation questionnaire

##### 2.1.1.1 Literature review

Systematic searches were conducted across PubMed, Web of Science, China National Knowledge Infrastructure (CNKI), and China Biology Medicine (CBM) databases from their inception to May 8, 2024. Table 1 presents the search strategies and retrieval results for each database. Supplementary manual searches of organizational websites—including the World Health Organization (WHO), the World Federation for Hospital Sterilization Sciences (WFHSS), and the Association of periOperative Registered Nurses (AORN)—yielded three additional relevant documents.

**Table 1:** Search database and search method.

Database	Search strategy	Quantity
Web of Science	(“Sterile Processing Department” (TS) or “Sterile Services Department” (TS) or “central supply room” (TS) or “central sterile supply department” (TS)) AND (education (TS) or training (TS) or curriculum (TS))	33
Pubmed	((((“Sterile Processing Department”) OR (“Sterile Services Department”)) OR (“central supply room”)) OR (“central sterile supply department”)) AND (((education) OR (training)) OR (curriculum))	55
China National Knowledge Infrastructure (CNKI)	(TI: Disinfection Supply Center (accurate)) OR (TI: Supply room (accurate)) AND (TKA: Training (accurate))	1,113
Sinomed	(“training “[All field: intelligent]) AND (“disinfection supply center/TI: intelligent” OR “Supply room”/TI: intelligent)	1,037

The following inclusion criteria were applied for literature selection: (1) studies focusing on training, education, curriculum development, or competency frameworks for CSSD personnel or sterile processing staff; (2) peer-reviewed articles, official guidelines, or government-issued standards; (3) publications in English or Chinese; (4) full-text availability. Exclusion criteria were: (1) studies unrelated to CSSD education or training (e.g., purely technical or engineering-focused studies without educational components); (2) conference abstracts, editorials, commentaries, or opinion pieces without original data or formal recommendations; (3) duplicate publications; (4) articles for which full text could not be obtained after requesting through interlibrary loan.

**Figure 1:**
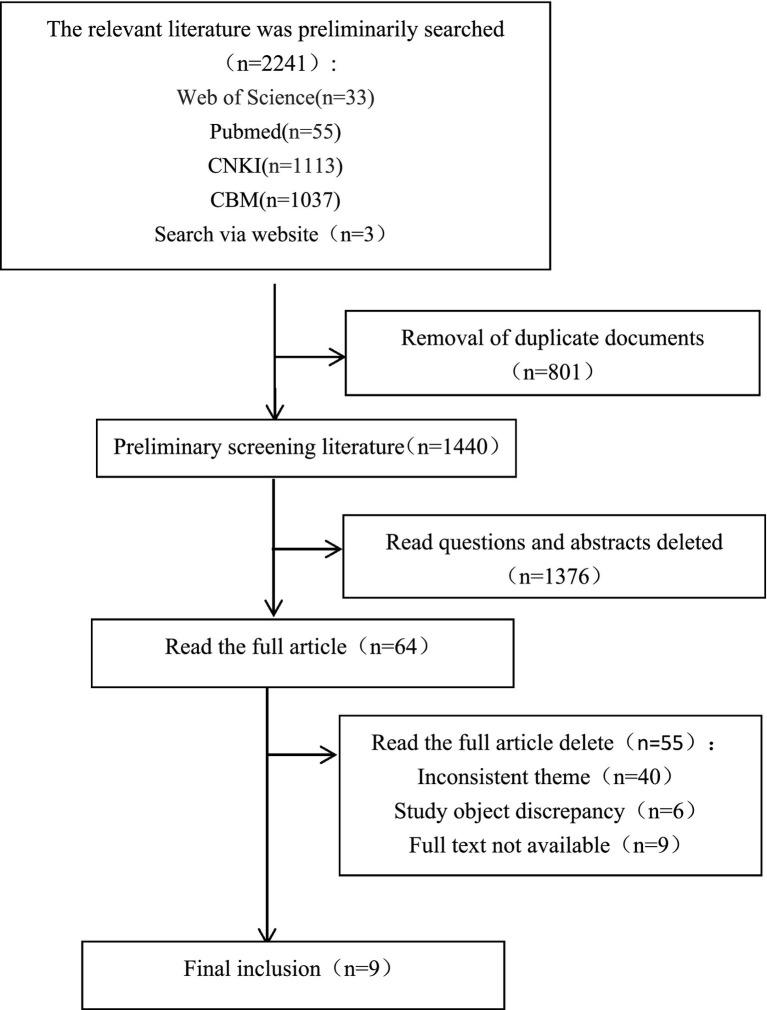
Flow chart of study selection.

A flow diagram (Figure 1) illustrates the screening process: initial retrieval (*n* = 2,241), removal of duplicates (*n* = 801), title and abstract screening (*n* = 1,440, with 1,376 excluded), full-text review (*n* = 64), and final inclusion (*n* = 9) (1, 10, 21–27). The specific exclusion reasons are detailed (inconsistent theme: *n* = 40; study object discrepancy: *n* = 6; full text unavailable: *n* = 9).

##### 2.1.1.2 Data extraction and synthesis

A standardized extraction instrument captured four domains from each included article: bibliographic information, study design, training process elements, training content domains. Two reviewers (LJW and GFL) independently extracted all data; discrepancies were resolved through discussion with arbitration by a third reviewer (CH) when needed. Extracted items were inductively coded into preliminary themes and aggregated into two frameworks: (i) training process and (ii) training content, yielding a draft indicator pool. This pool was presented to the expert panel for review and refinement before questionnaire formatting.

##### 2.1.1.3 Expert panel discussion

Experts were recruited through purposive sampling; the optimal panel size for Delphi studies is typically 10–15 participants (20). We convened a panel comprising five department heads, three clinical educators, and two infection control specialists (*n* = 10) to evaluate the preliminary indicator framework. Panelists assessed the clarity and comprehensiveness of indicators through structured discussion until saturation was achieved (i.e., no new indicators emerged). Incorporating panel feedback, we refined the framework to produce two preliminary index systems: a training process system (5 primary, 12 secondary, and 46 tertiary indicators) and a training content system (4 primary, 22 secondary, and 97 tertiary indicators) (see Supplementary file S1).

#### 2.1.2 Phase 2: Delphi consulting and feedback cycle

Delphi panelists were recruited through purposive sampling from nursing education, clinical management, infection control, and CSSD operations. Eligibility criteria included: (1) a minimum 5 years of relevant experience; (2) an intermediate or senior professional title; (3) a bachelor’s degree or higher; and (4) willingness to participate. Delphi methodology recommends a panel of 10–15 participants (28); however, to ensure independent perspectives, we excluded the 10 experts involved in the preliminary framework development and recruited 25 specialists from nine provinces and municipalities: Beijing, Chongqing, Gansu, Henan, Jiangsu, Shaanxi, Shanxi, Sichuan, and Zhejiang.

### 2.2 Data collection

Two rounds of questionnaires were distributed via email or WeChat between April and June 2024, with participants given 2 weeks for completion. Each questionnaire comprised four sections: (a) Introduction: study purpose, content overview, informed consent, and completion instructions; (b) Demographics: age, gender, education, professional title, and specialty area; (c) Indicator Rating: training process and content indicators rated on a 5-point Likert scale (1 = not important/not feasible, 5 = very important/highly feasible), with open-ended fields for additional indicators and revision suggestions; and (d) Authority Assessment: familiarity coefficient (Cs) rated from 0.20 (unfamiliar) to 1.00 (very familiar), and judgment coefficient (Ca) based on practical experience, theoretical analysis, peer consultation, and personal intuition, each weighted by self-rated reliance (high/moderate/low). Following first-round analysis, the research team revised the second-round instrument based on expert feedback (see Supplementary file S2). In Round 2, participants received controlled feedback comprising aggregated first-round results and anonymized comments from other panelists, enabling iterative refinement while maintaining strict anonymity through individualized distribution.

### 2.3 Data analysis

Data were analyzed using SPSS version 29.0 (IBM Corp, Armonk, NY, United States). Descriptive statistics included means, coefficients of variation (CV), and endorsement rates. Consensus thresholds were defined as: mean ≥ 3.5, CV < 0.25, and endorsement rate ≥ 70% (28). Consensus level was operationalized as the percentage of ratings at the maximum score (5/5). Three metrics assessed expert consultation quality: (1) response rate, indicating panel engagement; (2) authority coefficient (Cr), computed as (Cs + Ca)/2; and (3) Kendall’s W, measuring inter-rater agreement (range 0–1) (29).

### 2.4 Ethical considerations

This study was approved by the Biomedical Ethics Committee of West China Hospital, Sichuan University (Approval No. 2024-639) and conducted in accordance with the Declaration of Helsinki. Participants were informed of the study purpose prior to enrollment, and written informed consent was obtained. Participants retained the right to withdraw at any time without penalty.

## 3 Results

### 3.1 Demographic characteristics

In Round 1, 25 of the 27 distributed questionnaires were returned as valid (response rate: 92.6%). Round 2 achieved a 100% retention rate (25/25). Expert feedback was provided by 52% of panelists (13/25) in Round 1 and 24% (6/25) in Round 2. Table 2 presents the demographic characteristics of the expert panel.

**Table 2:** Expert demographic data.

Characteristics	Frequency (*N*)	Proportion (%)
Gender		
Men	1	4.00%
Women	24	96.00%
Age (years)		
<40	6	24.00%
40–50	8	32.00%
≥50	11	44.00%
Title		
Junior level		
Intermediate level	10	40.00%
Advanced level	15	60.00%
Education level		
Bachelor	18	72.00%
Master	6	24.00%
Doctor	1	4.00%
Work direction		
Nursing education	5	20.00%
Nursing management	6	24.00%
CSSD	12	48.00%
Nosocomial infection	2	8.00%
Work experience (years)		
<15	6	24.00%
15–25	13	52.00%
≥25	6	24.00%

### 3.2 Reliability of the expert panel

The Cr score for the two Delphi rounds was 0.912, indicating an acceptable level of expert authority (17). As shown in Table 3, Kendall’s W values ranged from 0.18 to 0.28 (*p* < 0.001), suggesting a moderate degree of consensus among the panel. This modest level of agreement is understandable given the large number of indicators and the comprehensive scope of the curriculum, which spans both training processes and content dimensions.

**Table 3:** Importance degree of coordination among experts.

Category	Training process				Training content			
	Items (*n*)	Kendall’s W	*X* ^2^	*p*-value	Items (*n*)	Kendall’s W	*X* ^2^	*p*-value
Round 1								
Total	63	0.199	333.835	<0.001	123	0.187	614.724	<0.001
Primary index	5	0.209	22.525	<0.001	4	0.250	20.270	<0.001
Secondary index	12	0.115	34.160	<0.001	22	0.177	100.337	<0.001
Three-level index	46	0.219	265.856	<0.001	97	0.188	486.630	<0.001
Round 2								
Total	67	0.227	380.660	<0.001	135	0.218	787.295	<0.001
Primary index	5	0.259	27.951	<0.001	4	0.282	22.816	<0.001
Secondary index	12	0.229	68.128	<0.001	24	0.273	169.491	<0.001
Three-level index	50	0.238	315.265	<0.001	107	0.201	575.079	<0.001

### 3.3 Summary of expert opinions

#### 3.3.1 Delphi round 1

In the first round, 13 experts proposed 27 amendments. The items “published more than 3 core journal articles in the past three years or one SCI article,” “dual mentorship in clinical training (professional mentor + research mentor),” and “writing review papers” had coefficients of variation greater than 0.25, while the rest reached an acceptable level of consensus (mean: 3.88–5; CV: 0–0.23; CLV: 72–100.00%). The research team reviewed the experts’ opinions and adjusted the index system to align with the research objectives. Based on expert suggestions, 13 items were modified, 4 were deleted, 2 were relocated, and 20 were added. All the retained and new index systems were sent to 25 experts for the second round of Delphi.

#### 3.3.2 Delphi round 2

In the second round, consensus was reached on 67 training process items and 135 training content indicators (mean values: 4.52–5.0; CV: 0–0.18; CLV: 72–100%). The experts proposed six revisions, including changing “1.2.1 Good health, passion for and recognition of work in the disinfection supply center, and a spirit of dedication” to “1.2.1 Good health without infectious diseases, passion for and recognition of work in the disinfection supply center, and a spirit of dedication,” and adding “2.2.7 Mastering emergency handling of CSSD incidents” to the training content indicators. Other changes included changing “2.7.2 Selection and application of packaging methods” to “Principles of packaging, prepackaging assessment, packaging operations,” “1.5 Knowledge of disinfection supply quality management,” “3.10 CSSD quality control methods and key points,” and “1.4.4 Knowledge related to sterilization media.” After discussion by the research team, these changes were not implemented because they either fell outside the scope of this study or were already reflected in existing indicators, making them redundant. At this point, the specialized nurse training system for the CSSD was fully completed, including the training process (5 primary indicators, 12 secondary indicators, and 50 tertiary indicators) and the training content (4 primary indicators, 24 secondary indicators, and 107 tertiary indicators). Detailed indicator information is available in the online Supplementary material: Expert Consultation Results of the Training System for Specialized Nurses in CSSD.^1^

## 4 Discussion

To our knowledge, this study represents the first consensus-based framework for CSSD specialist nurse training in China. Using a two-round modified Delphi approach, we established competency standards encompassing trainee selection, faculty qualifications, instructional modalities, program duration, and required competencies (knowledge, skills, attitudes, and professional attributes).

The resulting training framework demonstrates strong validity and reliability. First, the expert panel comprised 25 multidisciplinary specialists collectively representing nursing education, clinical management, infection prevention, and CSSD, ensuring comprehensive domain coverage and high authority (Cr = 0.912). Second, response rates of 92.6 and 100% reflect robust panel engagement. Third, two Delphi Cr scores exceeded 0.70, and two Kendall’s W scores showed statistical significance (*p* < 0.05). Although the Kendall’s W coefficients were relatively modest, they are comparable to those reported in other Delphi studies within nursing education that have addressed complex, multi-dimensional indicator systems. The statistical significance of these values, coupled with high response rates and rich qualitative input from experts, suggests that the consensus achieved—while moderate—is sufficient for establishing a foundational framework.

This study developed a comprehensive competency framework for CSSD specialist nurse training, encompassing five core domains and 50 specific performance indicators. Grounded in adult learning principles—which emphasize workplace-relevant knowledge and skills to enhance clinical efficiency (30, 31)—we specified a two-month, 320-h practicum based on expert consensus. Recognizing that instructional quality hinges on faculty expertise, we established rigorous qualifications for trainers across five dimensions: professional specialty, years of CSSD experience, teaching competency, institutional affiliation, and professional attributes. The evaluation system integrates formative process assessment with summative measures of theoretical knowledge, clinical competency, and teaching performance, offering a robust, multi-method approach to trainee assessment.

The training content framework developed in this study is systematic, evidence-based, and feasible. Experts evaluated indicator importance across four domains—knowledge, technical skills, professional competencies, and personal attributes—and provided supplementary feedback, ensuring comprehensive coverage and practical applicability. Both rounds of consultation revealed strong expert emphasis on “infection prevention and occupational protection.” This priority reflects the central role of CSSD in hospital-acquired infection control and aligns with international guidelines mandating personal protective measures across all CSSD operations (1, 10). Despite these standards, evidence indicates persistent gaps in CSSD occupational safety (32), underscoring the necessity of dedicated training in this domain.

Additionally, rapid advances in surgical instrumentation and diagnostic technologies have complicated device reprocessing requirements. Ensuring timely delivery of properly sterilized instruments is critical for patient safety and clinical service quality. Information technology offers substantial potential for enhancing CSSD efficiency; however, implementation challenges persist, including IT specialists’ limited understanding of CSSD workflows and managers’ inadequate technical proficiency (33). To address this competency gap, “health informatics” has been integrated into the training framework to support digital transformation in healthcare facilities.

Finally, CSSD operations are resource-intensive, characterized by high capital investment and material consumption. Balancing cost containment with quality assurance remains an ongoing management challenge (34). Consequently, ‘resource and cost management’ has been incorporated to equip practitioners with essential financial stewardship skills.

While the indicator system encompasses over 107 tertiary items to ensure comprehensive coverage across diverse CSSD functions, we acknowledge that complete implementation may not be immediately feasible in all settings. We recommend a modular, stepwise adoption approach, wherein institutions prioritize indicators most relevant to their current operational scope and gradually expand training components as resources and capacity permit. This flexible implementation strategy balances the need for thorough training with the practical realities of clinical workload constraints.

## 5 Future directions

To extend the applicability of this training curriculum beyond China, future research should prioritize cross-cultural adaptation and validation through a three-pronged approach. First, engage expert panels from diverse healthcare systems—such as those in North America, Europe, and the Asia-Pacific region—to review and contextualize the indicators according to local regulations and professional scopes of practice. Second, conduct international Delphi studies or consensus conferences to pinpoint core competencies that transcend national boundaries. Third, pilot-test the adapted curriculum across multiple countries to assess both cross-cultural feasibility and effectiveness, measuring outcomes through pre- and post-intervention evaluations of nurse competence using validated instruments, alongside longitudinal tracking of infection-related metrics where possible. Such efforts would not only yield empirical evidence of the curriculum’s educational and clinical impact but also significantly bolster the framework’s global generalizability.

## 6 Limitations

It is important to recognize certain constraints present in this research. First, despite specialists hailing from nine distinct provinces across China’s three primary economic regions, regional bias may still exist. Moreover, the geographic concentration of our expert panel represents a notable limitation—all 25 experts were recruited from China, meaning that the developed training index system is inherently shaped by the Chinese healthcare context, including its specific regulations, educational norms, and clinical practices. Therefore, caution is warranted when attempting to transfer or apply this curriculum directly to other countries or healthcare systems with different cultural, regulatory, or resource contexts. Second, although the number of experts was acceptable and the panel was authoritative, the fact that nearly 50% specialized in disinfection materials suggests that the final index system may not fully represent an educational perspective. Future research should incorporate broader input from educational specialists to refine the training index system. Third, while the Delphi method ensured content validity through expert consensus, this study did not proceed to empirical validation. Consequently, the actual impact of this curriculum on improving nurse competence, compliance behaviors, or patient infection outcomes remains unknown—representing the most significant limitation of this work.

## 7 Conclusion

Through systematic literature review, expert consultation, and two-round Delphi consensus, this study developed a training framework for CSSD specialist nurses. The framework balances individual competency development with the operational realities of training institutions. Grounded in evidence-based indicators and expert validation, the framework demonstrates strong validity and practical feasibility. It offers actionable guidance for faculty recruitment and qualification standards in CSSD specialist programs, establishing a foundation for standardized training in the specialty.

## Data Availability

The original contributions presented in the study are included in the article/Supplementary material, further inquiries can be directed to the corresponding author.

## Footnotes

1
https://docs.qq.com/doc/DS0Nub1FneGVhZXRn
