# Supplementary material for: Achieving consensus on the curriculum system for central sterile supply department nurses: a modified Delphi study
Source: Front Med (Lausanne). 2026 May 7;13:1774004. doi: 10.3389/fmed.2026.1774004 (PMC13189882; doi:10.3389/fmed.2026.1774004)
Supplement: Supplementary file 1 [file Table_1.DOCX]

**Construction of the Training System for Specialized Nurses in the CSSD: Expert Consultation Form (First Round)**

Dear Expert,

Greetings!

We sincerely invite you to participate in this Delphi expert survey, in recognition of your valuable expertise. Thank you very much for taking the time to complete this questionnaire despite your busy schedule.

The aim of this study is to develop a specialized training system for nurses in the Central Sterile Supply Department (CSSD), thereby providing a scientific basis for further enhancing and improving the core competencies of CSSD nurses.

Based on a thorough review and synthesis of relevant literature and materials on CSSD specialized nursing both domestically and internationally, we have summarized, organized, and discussed the relevant indicator frameworks, leading to the development of the proposed indicator system presented in this survey. The purpose of this expert consultation is to gather opinions from professionals in the fields of disinfection supply, nursing management, hospital administration, infection control, and related areas, in order to finalize a training indicator system for CSSD specialized nurses. It is anticipated that two to three rounds of correspondence surveys will be conducted.

Your input is critically important to this research. Please complete the questionnaire according to the instructions. There are no right or wrong answers — we are interested only in your genuine opinions.

This questionnaire consists of three parts:

Part 1: Background information of the expert.

Part 2: Delphi questionnaire on the indicator system, including:

(1) Construction of the training process indicator system for CSSD specialized nurses;

(2) Construction of the training content indicator system for CSSD specialized nurses.

You will be asked to rate each indicator based on its importance.

Part 3: Expert analysis, aimed at understanding your familiarity and basis for judgment regarding the indicators.

Given the time constraints of this study, we would greatly appreciate it if you could complete the questionnaire at your earliest convenience.

Thank you once again for participating in this research. We sincerely welcome any suggestions or feedback you may have regarding this study.

With kind regards and respect,

CSSD, West China Hospital, Sichuan University
April 2024

**Part 1: Expert Background Information**

1. ****Name (optional)****：
2. ****Gender:**** □ Male □ Female
3. ****Year of birth:**** :
4. ****Institution/affiliation:****
5. ****Position title:**** ：
6. ****Professional title****：

( ) A. Junior( ) B. Intermediate( ) C. Associate senior( ) D. Senior

1. ****Highest educational degree:****

( ) A. Associate degree( ) B. Bachelor's degree( ) C. Master's degree( ) D. Doctoral degree

1. ****Your current primary role(s) (multiple selections allowed):****（ ）（多选）

(Please indicate the number of years of experience next to each selected role.)

( ) A. Central Sterile Supply Department (CSSD) operations ______ years

( ) B. Nursing management ______ years
( ) C. Hospital administration/management ______ years

( ) D. Nursing education ______ years

( ) E. Other (please specify): _________________ ______ years

**Part 2: Construction of the Training System for Specialized Nurses in the Central Sterile Supply Department (CSSD)**

Instructions:

1.In this survey, each indicator is evaluated based on two dimensions: importance and feasibility, to determine the appropriateness of the selected indicators.Importance refers to the significance of the indicator.

You may add, delete, or modify indicators as you see fit.

(1)If you believe an indicator should be added, please write the suggested content in the "Suggested Additions" column.

(2)If you believe an indicator should be deleted or modified, please provide your comments and reasons in the "Modification Suggestions" column next to the corresponding indicator.

3.A five-point Likert scale is used to rate the importance and feasibility of each indicator. Importance: Scores range from 1 to 5, where 5 indicates the indicator is most important and 1 indicates it is least important.

(2)Please mark your score with a "√" in the corresponding box.

## ****Part 2.1:**Construction of the Training Process Indicator System for CSSD Specialized Nurses**

## Table 1-1. Level 1 indicators of the CSSD specialized nurse training process system

| **Level 1 indicators** | Importance | | | | | | Modification Suggestions | |
| --- | --- | --- | --- | --- | --- | --- | --- | --- |
|  | **5** | **4** | **3** | **2** | **1** |  | |  |
| 1.Admission requirements |  |  |  |  |  |  | |  |
| 2.Training objectives |  |  |  |  |  |  | |  |
| **3.Trainer qualifications** |  |  |  |  |  |  | |  |
| **4.Training methods and duration** |  |  |  |  |  |  | |  |
| 5.Evaluation focus |  |  |  |  |  |  | |  |
| Suggested Additions |  | | | | | | |  |

**Table 1-2. Level 2 indicators of the CSSD specialized nurse training process system**

| **Level 1 indicators** | **Level 2 indicators** | Importance | | | | | Modification Suggestions |
| --- | --- | --- | --- | --- | --- | --- | --- |
|  |  | 5 | 4 | 3 | 2 | 1 |  |
| 1. Admission requirements | 1.1 Basic conditions |  |  |  |  |  |  |
|  | 1.2 Literacy conditions |  |  |  |  |  |  |
| Suggested Additions | |  | | | | | |
| 2. Training objectives | 2.1 Literacy goals |  |  |  |  |  |  |
|  | 2.2 Knowledge objectives |  |  |  |  |  |  |
|  | 2.3 Skill objectives |  |  |  |  |  |  |
| Suggested Additions | |  | | | | | |
| 3. Trainer qualifications | 3.1 Basic conditions |  |  |  |  |  |  |
|  | 3.2 Literacy conditions |  |  |  |  |  |  |
| Suggested Additions | |  | | | | | |
| 4. Training method and time | 4.1 Theoretical training methods |  |  |  |  |  |  |
|  | 4.2 Practical training methods |  |  |  |  |  |  |
|  | 4.3 Training time |  |  |  |  |  |  |
| Suggested Additions | |  | | | | | |
| 5. Evaluation focus | 5.1 Training process assessment |  |  |  |  |  |  |
|  | 5.2 Training completion assessment |  |  |  |  |  |  |
| Suggested Additions | |  | | | | | |

**Table 1-3. Level 3 indicators of the CSSD specialized nurse training process system**

| **Level 1 indicators** | **Level 2 indicators** | **Leve3 indicators** | importance | | | | | Modification Suggestions |
| --- | --- | --- | --- | --- | --- | --- | --- | --- |
|  |  |  | 5 | 4 | 3 | 2 | 1 |  |
| 1. Admission requirements | 1.1 Basic conditions | 1.1.1 College degree or above |  |  |  |  |  |  |
|  |  | 1.1.2 Have the nurse practice certificate, nurse title or above. |  |  |  |  |  |  |
|  |  | 1.1.3 Working in this major for more than 3 years |  |  |  |  |  |  |
|  |  | 1.1.4 My application and recommendation by the hospital. |  |  |  |  |  |  |
|  |  | Suggested Additions |  | | | | | |
|  | 1.2 Literacy conditions | 1.2.1 Good health, love and identify with the disinfection supply center work, and dedication. |  |  |  |  |  |  |
|  |  | 1.2.2 Observe the discipline and respect the teaching teachers. |  |  |  |  |  |  |
|  |  | Suggested Additions |  | | | | | |
| 2. Training objectives | 2.1 Literacy goals | 2.1.1 Have the spirit of prudence and treat daily work with a rigorous attitude. |  |  |  |  |  |  |
|  |  | 2.1.2 Keep a positive and optimistic attitude and conduct self-debugging to meet the team development. |  |  |  |  |  |  |
|  |  | 2.1.3 Pay attention to the professional development trends, and constantly improve yourself. |  |  |  |  |  |  |
|  |  | 2.1.4 Compliance with ethical and legal and regulatory requirements |  |  |  |  |  |  |
|  |  | Suggested Additions |  | | | | | |
|  | 2.2 Knowledge objectives | 2.2.1 Master the CSSD infection prevention and quality control |  |  |  |  |  |  |
|  |  | 2.2.2 Master the working principle of the CSSD equipment |  |  |  |  |  |  |
|  |  | 2.2.3 Master the performance and management requirements of reusable surgical instruments |  |  |  |  |  |  |
|  |  | Suggested Additions |  | | | | | |
|  | 2.3 Skill objectives | 2.3.1 Master the reprocessing process of specialized surgical instruments |  |  |  |  |  |  |
|  |  | 2.3.2 Master the daily use and maintenance of the equipment and facilities |  |  |  |  |  |  |
|  |  | 2.3.3 Master the principles and skills of multi-department communication |  |  |  |  |  |  |
|  |  | 2.3.4 Ability to carry out scientific research and teaching |  |  |  |  |  |  |
|  |  | Suggested Additions |  | | | | | |
| 3. Trainer qualifications | 3.1 Basic conditions | 3.1.1 Bachelor degree or above |  |  |  |  |  |  |
|  |  | 3.1.2 Title of associate senior or above |  |  |  |  |  |  |
|  |  | 3.1.3 Obtain relevant professional qualification certificates |  |  |  |  |  |  |
|  |  | 3.1.4 Published more than 3 core journals or more than one SCI article in the past three years |  |  |  |  |  |  |
|  |  | 3.1.5 The major is elimination / hospital awareness / management / teaching / scientific research / endoscopy. |  |  |  |  |  |  |
|  |  | 3.1.6 More than 10 years of relevant professional working experience |  |  |  |  |  |  |
|  |  | 3.1.7 More than 5 years of teaching experience. |  |  |  |  |  |  |
|  |  | 3.1.8 Working units are institutions directly under the province. |  |  |  |  |  |  |
|  |  | Suggested Additions |  | | | | | |
|  | 3.2 Literacy conditions | 3.2.1 Love my major and love my teaching work. |  |  |  |  |  |  |
|  |  | 3.2.2 Past students have not put forward negative opinions against medical ethics and teacher quality. |  |  |  |  |  |  |
|  |  | Suggested Additions |  | | | | | |
| 4. Training methods and duration | 4.1 Theoretical training methods | 4.1.1 Multimedia teaching |  |  |  |  |  |  |
|  |  | 4.1.2 Work visit |  |  |  |  |  |  |
|  |  | 4.1.3 Case analysis group discussion |  |  |  |  |  |  |
|  |  | 4.1.4 PBL teaching |  |  |  |  |  |  |
|  |  | 4.1.5 Flipped classroom |  |  |  |  |  |  |
|  |  | Suggested Additions |  | | | | | |
|  | 4.2 Practical training methods | 4.2.1 Clinical one-site teaching |  |  |  |  |  |  |
|  |  | 4.2.2 Clinical double tutorial system (professional supervisor + scientific research supervisor) |  |  |  |  |  |  |
|  |  | 4.2.3 Scenario simulation teaching |  |  |  |  |  |  |
|  |  | 4.2.4 Operation instruction |  |  |  |  |  |  |
|  |  | Suggested Additions |  | | | | | |
|  | 4.3 Training duration | 4.3.1 The theoretical training lasts one month and the total duration is 160 hours. |  |  |  |  |  |  |
|  |  | 4.3.2 The practical training lasts for one month with a total of 160 hours. |  |  |  |  |  |  |
|  |  | Suggested Additions |  | | | | | |
| 5. Evaluation focus | 5.1 Process assessment | 5.1.1 Medical ethics |  |  |  |  |  |  |
|  |  | 5.1.2 Professional Literacy |  |  |  |  |  |  |
|  |  | 5.1.3 Daily performance (attendance) |  |  |  |  |  |  |
|  |  | 5.1.4 Stage completion situation |  |  |  |  |  |  |
|  |  | Suggested Additions |  | | | | | |
|  | 5.2 Completion assessment | 5.2.1 Assessment of theoretical knowledge |  |  |  |  |  |  |
|  |  | 5.2.2 Assessment of practical skills |  |  |  |  |  |  |
|  |  | 5.2.3 Writing of the review paper |  |  |  |  |  |  |
|  |  | 5.2.4 Small lectures |  |  |  |  |  |  |
|  |  | Suggested Additions |  | | | | | |

**Part2.2:Construction of the Training Content Indicator System for Specialized Nurses in the Central Sterile Supply Department** Table 2-1 .Level 1 indicators of the CSSD specialized nurse training content indicator system

| **Level 1 indicators** | importance | | | | | Modification Suggestions |
| --- | --- | --- | --- | --- | --- | --- |
|  | 5 | 4 | 3 | 2 | 1 |  |
| 1. knowledge |  |  |  |  |  |  |
| 2. Skills |  |  |  |  |  |  |
| 3. Ability |  |  |  |  |  |  |
| 4. Feature |  |  |  |  |  |  |
| Suggested Additions |  | | | | | |

Table 2-2. Leve 2 indicators of the CSSD specialized nurse training content indicator system

| **Level 1 indicators** | **Level 2 indicators** | importance | | | | | Modification Suggestions |
| --- | --- | --- | --- | --- | --- | --- | --- |
|  |  | 5 | 4 | 3 | 2 | 1 |  |
| 1. knowledge | 1.1 Overview of the disinfection supply center |  |  |  |  |  |  |
|  | 1.2 Basic knowledge related to disinfection supply |  |  |  |  |  |  |
|  | 1.3 Knowledge of disinfection and sterilization |  |  |  |  |  |  |
| Suggested Additions | |  | | | | | |
| 2. Skills | 2.1 Preprocessing technology |  |  |  |  |  |  |
|  | 2.2 Device recovery technology |  |  |  |  |  |  |
|  | 2.3 Device classification technique |  |  |  |  |  |  |
|  | 2.4 Device cleaning and disinfection technology |  |  |  |  |  |  |
|  | 2.5 Device Drying technique |  |  |  |  |  |  |
|  | 2.6 Device inspection and maintenance technology |  |  |  |  |  |  |
|  | 2.7 Device packaging technology |  |  |  |  |  |  |
|  | 2.8 Device Sterilization technique |  |  |  |  |  |  |
|  | 2.9 Device storage and distribution |  |  |  |  |  |  |
|  | 2.10 Logistics and distribution of devices |  |  |  |  |  |  |
|  | 2.11 Quality monitoring technology |  |  |  |  |  |  |
| Suggested Additions | |  | | | | | |
| 3. Ability | 3.1 Management ability |  |  |  |  |  |  |
|  | 3.2 Risk management and control ability |  |  |  |  |  |  |
|  | 3.3 Learning ability |  |  |  |  |  |  |
|  | 3.4 Scientific research ability |  |  |  |  |  |  |
|  | 3.5 Teaching ability |  |  |  |  |  |  |
|  | 3.6 Communication skills |  |  |  |  |  |  |
|  |  |  |  |  |  |  |  |
| 4. Feature | 4.1 Code of professional ethics |  |  |  |  |  |  |
|  | 4.2 Personal literacy |  |  |  |  |  |  |
| Suggested Additions | |  | | | | | |

Table 2-3. Leve 3indicators of the CSSD specialized nurse training content indicator system

| **Level 1 indicators** | **Level 2 indicators** | **Level 3 indicators** | importance | | | | | | | | | Modification Suggestions |
| --- | --- | --- | --- | --- | --- | --- | --- | --- | --- | --- | --- | --- |
|  |  |  | 5 | | 4 | | 3 | | | 2 | 1 |  |
| 1. knowledge | 1.1 Overview of the disinfection supply center | 1.1.1 Development course of CSSD at home and abroad |  | |  | |  | | |  |  |  |
|  |  | 1.1.2 Management mode of CSSD |  | |  | |  | | |  |  |  |
|  |  | 1.1.3 Building and layout requirements of CSSD |  | |  | |  | | |  |  |  |
|  |  | 1.1.4 Job responsibilities and system of CSSD |  | |  | |  | | |  |  |  |
|  |  | 1.1.5 Relevant laws and regulations and industry standards of CSSD |  | |  | |  | | |  |  |  |
|  | Suggested Additions | |  | | | | | | | | | |
|  | 1.2 Basic knowledge related to disinfection supply | 1.2.1 Basic knowledge of medical devices |  | |  | |  | | |  |  |  |
|  |  | 1.2.2 Basic principles of CSSD equipment and facilities |  | |  | |  | | |  |  |  |
|  | Suggested Additions | |  | | | | | | | | | |
|  | 1.3 Knowledge of disinfection and sterilization | 1.3.1 Common microbial species |  | |  | |  | | |  |  |  |
|  |  | 1.3.2 Common methods for disinfection and sterilization |  | |  | |  | | |  |  |  |
|  |  | 1.3.3 Infection prevention and occupational protection in CSSD |  | |  | |  | | |  |  |  |
|  |  | 1.3.4Management of the occupational exposure |  | |  | |  | | |  |  |  |
|  | Suggested Additions | |  | |  | |  | | |  |  |  |
| 2. Skills | 2.1 Preprocessing technology | 2.1.1 Treatment on site |  | |  | |  | | |  |  |  |
|  |  | 2.1.2 Pretreatment before cleaning |  | |  | |  | | |  |  |  |
|  | Suggested Additions | |  | |  | |  | | |  |  |  |
|  | 2.2 Device recovery technology | 2.2.1 Recovery of conventional devices |  | |  | |  | | |  |  |  |
|  |  | 2.2.2 Recovery of specialized precision equipment |  | |  | |  | | |  |  |  |
|  |  | 2.2.3 Recovery of special contaminated devices |  | |  | |  | | |  |  |  |
|  |  | 2.2.4 Recovery of implants and external medical devices |  | |  | |  | | |  |  |  |
|  |  | 2.2.5 Recovery of soft endoscopic instruments and hard endoscopic instruments |  | |  | |  | | |  |  |  |
|  |  | 2.2.6 Recovery of cross-hospital devices |  | |  | |  | | |  |  |  |
|  |  | 2.2.7 Disposal of recycling tools |  | |  | |  | | |  |  |  |
|  | Suggested Additions | |  | | | | | | | | | |
|  | 2.3 Device classification technique | 2.3.1 Classification according to the device material |  | |  | |  | | |  |  |  |
|  |  | 2.3.2 Classification according to the device structure |  | |  | |  | | |  |  |  |
|  |  | 2.3.3 Classification according to the degree of device contamination |  | |  | |  | | |  |  |  |
|  |  | 2.3.4 Classification according to the device for heat resistance and moisture resistance |  | |  | |  | | |  |  |  |
|  | Suggested Additions | |  | | | | | | | | | |
|  | 2.4 Device cleaning and disinfection technology | 2.4.1 Manual cleaning techniques (including cleaning of soft endoscope, robotic surgical instruments, endoscopic instruments, etc.) |  | |  | |  | | |  |  |  |
|  |  | 2.4.2 Mechanical cleaning technology |  | |  | |  | | |  |  |  |
|  |  | 2.4.3 Selection of cleaning agent and disinfectant |  | |  | |  | | |  |  |  |
|  |  | 2.4.4 Configuration of cleaning agent and disinfectant |  | |  | |  | | |  |  |  |
|  |  | 2.4.5 Cleaning and disinfection process of special infected devices |  | |  | |  | | |  |  |  |
|  |  | 2.4.6 Use and daily maintenance of ultrasonic cleaning machine |  | |  | |  | | |  |  |  |
|  |  | 2.4.7 Use and daily maintenance of mechanical cleaning machine |  | |  | |  | | |  |  |  |
|  |  | 2.4.8 Use and daily maintenance of water treatment equipment |  | |  | |  | | |  |  |  |
|  | Suggested Additions | |  | |  | |  | | |  |  |  |
|  | 2.5 Device Drying technique | 2.5.1 Selection of drying mode |  | |  | |  | | |  |  |  |
|  |  | 2.5.2 Judgment of the drying effect |  | |  | |  | | |  |  |  |
|  |  | 2.5.3 Use and daily maintenance of drying equipment |  | |  | |  | | |  |  |  |
|  | Suggested Additions | |  | | | | | | | | | |
|  | 2.6 Device inspection and maintenance | 2.6.1 Inspection of the device cleanliness |  | |  | |  | | |  |  |  |
|  |  | 2.6.2 Check of the functional status of the device |  | |  | |  | | |  |  |  |
|  |  | 2.6.3 Insulation performance test of the device |  | |  | |  | | |  |  |  |
|  |  | 2.6.4 Disassembly and assembly of the device |  | |  | |  | | |  |  |  |
|  |  | 2.6.5 Maintenance technology of the devices |  | |  | |  | | |  |  |  |
|  | Suggested Additions | |  | | | | | | | | | |
|  | 2.7 Device packaging technology | 2.7.1 Selection of packaging materials |  | |  | |  | | |  |  |  |
|  |  | 2.7.2 Selection and application of packaging method |  | |  | |  | | |  |  |  |
|  |  | 2.7.3 Use and daily maintenance of medical sealing machine |  | |  | |  | | |  |  |  |
|  |  | 2.7.4 Application of information traceability system and label printing |  | |  | |  | | |  |  |  |
|  |  | 2.7.5 Inspection of packaging quality |  | |  | |  | | |  |  |  |
|  | Suggested Additions | |  | | | | | | | | | |
|  | 2.8 Device Sterilization technique | 2.8.1 The loading and unloading of sterilized articles |  | |  | |  | | |  |  |  |
|  |  | 2.8.2 Use and daily maintenance of pressure steam sterilizer |  | |  | |  | | |  |  |  |
|  |  | 2.8.3 Use and daily maintenance of ethylene oxide sterilizer |  | |  | |  | | |  |  |  |
|  |  | 2.8.4 Use and daily maintenance of hydrogen peroxide low-temperature plasma sterilizer |  | |  | |  | | |  |  |  |
|  |  | 2.8.5 Use and daily maintenance of low-temperature steam formaldehyde sterilizer |  | |  | |  | | |  |  |  |
|  |  | 2.8.6 Interpretation of sterilization results (physical, chemical, biological) |  | |  | |  | | |  |  |  |
|  | Suggested Additions | |  | | | | | | | | | |
|  | 2.9 Device storage and distribution | 2.9.1 Storage and validity period of sterile items |  | |  | |  | | |  |  |  |
|  |  | 2.9.2 Distribution process of sterile articles |  | |  | |  | | |  |  |  |
|  |  | 2.9.3 Distribution process of emergency delivery devices |  | |  | |  | | |  |  |  |
|  |  | Suggested Additions |  | | | | | | | | | |
|  | 2.10 Logistics and distribution of devices | 2.10.1 Device transfer of devices |  | |  | |  | | |  |  |  |
|  |  | 2.10.2 Hospital-level device transfer |  | |  | |  | | |  |  |  |
|  | Suggested Additions | |  | | | | | | | | | |
|  | 2.11 Quality monitoring technology | 2.11.1 Water quality monitoring |  | |  | |  | | |  |  |  |
|  |  | 2.11.2 Monitoring of the environment and staff hand hygiene in CSSD |  | |  | |  | | |  |  |  |
|  |  | 2.11.3 Monitoring technology of cleaning quality and interpretation of results |  | |  | |  | | |  |  |  |
|  |  | 2.11.4 Monitoring technology of sterilization quality and interpretation of results |  | |  | |  | | |  |  |  |
|  |  | 2.11.5 Recall and disposal process of unqualified items |  | |  | |  | | |  |  |  |
|  | Suggested Additions | |  | | | | | | | | | |
| 3. Ability | 3.1 Management ability | 3.1.1 CSSD material management |  | |  | |  | | |  |  |  |
|  |  | 3.1.2 CSSD organization and management |  | |  | |  | | |  |  |  |
|  |  | 3.1.3 Performance management of CSSD |  | |  | |  | | |  |  |  |
|  |  | 3.1.4 CSSD Cost management |  | |  | |  | | |  |  |  |
|  |  | 3.1.5 CSSD equipment and facilities management |  | |  | |  | | |  |  |  |
|  |  | 3.1.6 Adverse event management of CSSD |  | |  | |  | | |  |  |  |
|  |  | 3.1.7 CSSD information construction and management |  | |  | |  | | |  |  |  |
|  |  | 3.1.8 Management of relevant documents and records |  | |  | |  | | |  |  |  |
|  |  | 3.1.9 Use of common quality management tools |  | |  | |  | | |  |  |  |
|  | Suggested Additions | |  | | | | | | | | | |
|  | 3.2 Risk management and control ability | 3.2.1 Risk prevention capability |  | |  | |  | | |  |  |  |
|  |  | 3.2.2 Emergency control capability |  | |  | |  | | |  |  |  |
|  | Suggested Additions | |  | | | | | | | | | |
|  | 3.3 Learning ability | 3.3.1 Learning and application of new technology and new business |  | |  | |  | | |  |  |  |
|  |  | 3.3.2 Ability to obtain professional information |  | |  | |  | | |  |  |  |
|  | Suggested Additions | |  | | | | | | | | | |
|  | 3.4 Scientific research ability | 3.4.1 Literature retrieval and reading ability |  | |  | |  | | |  |  |  |
|  |  | 3.4.2 Scientific research topics |  | |  | |  | | |  |  |  |
|  |  | 3.4.3 Methods of scientific research and design |  | |  | |  | | |  |  |  |
|  |  | 3.4.4 Collection and management of scientific research data |  | |  | |  | | |  |  |  |
|  |  | 3.4.5 Common statistical methods |  | |  | |  | | |  |  |  |
|  |  | 3.4.6 Writing of the nursing paper |  | |  | |  | | |  |  |  |
|  |  | 3.4.7 New technology and new business development |  | |  | |  | | |  |  |  |
|  |  | 3.4.8 Project development and management |  | |  | |  | | |  |  |  |
|  |  | 3.4.9 Ability to disseminate scientific research achievements |  | |  | |  | | |  |  |  |
|  | Suggested Additions | |  | | | | | | | | | |
|  | 3.5 Teaching ability | 3.5.1 Organization and management of clinical teaching |  | |  | |  | | |  |  |  |
|  |  | 3.5.2 Clinical teaching methods |  | |  | |  | | |  |  |  |
|  |  | 3.5.3 Clinical teaching skills |  | |  | |  | | |  |  |  |
|  |  | 3.5.4 Evaluation of clinical teaching |  | |  | |  | | |  |  |  |
|  | Suggested Additions | |  | | | | | | | | | |
|  | 3.6 Communication skills | 3.6.1 Communication ability with the service department |  | |  | |  | | |  |  |  |
|  |  | 3.6.2 Communication ability with foreign consumer consumption service units |  | |  | |  | | |  |  |  |
|  |  | 3.6.3 Communication ability with the guarantee department |  | |  | |  | | |  |  |  |
|  |  | 3.6.4 Communication skills with the personnel in the department |  | |  | |  | | |  |  |  |
|  | Suggested Additions | |  | | | | | | | | | |
| 4. Feature | 4.1 Code of professional ethics | 4.1.1 Spirit of prudence |  |  | |  | |  |  | | |  |
|  |  | 4.1.2 Prevention of job burnout |  |  | |  | |  |  | | |  |
|  |  | 4.1.3 Empathy and responsibility |  |  | |  | |  |  | | |  |
|  | Suggested Additions | |  | | | | | | | | | |
|  | 4.2 Personal literacy | 4.2.1 Emotional management ability |  |  | |  | |  |  | | |  |
|  |  | 4.2.2 Suitability |  |  | |  | |  |  | | |  |
|  |  | 4.2.3 Decision-making ability |  |  | |  | |  |  | | |  |
|  | Suggested Additions | |  | | | | | | | | | |

Part 3: Expert Judgment Analysis

1.Please indicate the basis for your judgments when completing the expert consultation form for the indicators above. Place a "√" in the appropriate box(es) in the table below.

| **Basis for judgment** | **High** | **Medium** | **Low** |
| --- | --- | --- | --- |
| Theoretical analysis |  |  |  |
| Practical experience |  |  |  |
| Knowledge of peer practices |  |  |  |
| Personal intuition |  |  |  |

2.Please rate the level of influence of each basis for judgment on your decision-making when completing the expert consultation form above. Mark "√" in the corresponding box(es):

| ****Expert's familiarity with the management system content:**** | Very familiar | Moderately familiar | Somewhat familiar | Not very familiar | Not at all familiar |
| --- | --- | --- | --- | --- | --- |
|  |  |  |  |  |  |
