# Supplementary material for: Achieving consensus on the curriculum system for central sterile supply department nurses: a modified Delphi study
Source: Front Med (Lausanne). 2026 May 7;13:1774004. doi: 10.3389/fmed.2026.1774004 (PMC13189882; doi:10.3389/fmed.2026.1774004)
Supplement: Supplementary file 2 [file Table_2.DOCX]

**Construction of the Training System for Specialized Nurses in the CSSD: Expert Consultation Form (Second Round)**

**Dear experts:**

Greetings! First of all, thank you very much for the time and effort you invested in the previous round of the questionnaire.

Through group discussions and literature review, our research team initially developed a framework for a training system for specialized nurses in the Central Sterile Supply Department (CSSD). We hope that through this expert consultation (Delphi method), we can refine this training system and provide a useful reference for clinical practice.

Based on the first round of expert consultation, you and the other experts provided very valuable suggestions regarding the training system. In response to these opinions and after conducting relevant statistical analyses, we have improved and revised several indicators. Consequently, we have developed the second-round expert consultation questionnaire, which consists of two main parts:

Part 1: The training process indicator system (including 5 primary indicators, 12 secondary indicators, and 50 tertiary indicators).

Part 2: The training content indicator system (including 4 primary indicators, 24 secondary indicators, and 107 tertiary indicators).

Please note: The indicators revised based on the first-round expert opinions are marked in red.

We kindly ask for your continued guidance and support. Please provide your feedback and suggestions within two weeks (by May 26, 2024).

Thank you again for your invaluable support. We wish you all the best in your work and good health!

Sincerely,

CSSD, West China Hospital, Sichuan University

May, 2024

**Questionnaire Adjustment Instructions**

After thoroughly discussing the experts' valuable feedback and consulting relevant literature and materials, the research group has made certain adjustments to the questionnaire items. The adjustments are explained as follows:

Deleted items: Items that are no longer included in the second round of the questionnaire are not presented.

New items: new items are indicated with the word "New".

Modified items: Modified items are displayed in red font.

**Fill in the form instructions**

Based on the feedback received from the first round of expert consultation, the training system for specialized nurses in the Central Sterile Supply Department (CSSD) has been revised to form the second-round questionnaire. Items revised based on first-round expert comments are displayed in red font.

Please rate each indicator based on your own expertise in terms of importance using the five-point scoring method:

Importance: Scores range from 1 to 5, where 5 indicates the item is most important and 1 indicates it is least important.

Please mark "√" under the corresponding option.

If you believe an indicator should be deleted or modified, please provide your suggestions and reasons in the "Modification Comments" column. If you have additional items or other amendments, please add them in the column labeled "Suggested Additions (Other Modification Comments)".

## Part I: **Construction of the Training Process Indicator System for CSSD Specialized Nurses**

Table 1-1. Level 1 indicators of the CSSD specialized nurse training process system

| **Level 1 indicators** | **importance** | | | | | **Modification Comments** |
| --- | --- | --- | --- | --- | --- | --- |
|  | **5** | **4** | **3** | **2** | **1** |  |
| 1. Admission requirements |  |  |  |  |  |  |
| 2. Training objectives |  |  |  |  |  |  |
| 3. Trainer qualifications |  |  |  |  |  |  |
| 4. Training methods and duration |  |  |  |  |  |  |
| **5. Assessment and evaluation** |  |  |  |  |  |  |
| Suggested Additions |  | | | | | |

Table 1-2. Level 2 indicators of the CSSD specialized nurse training process system

| Level 1 indicators | Level 2 indicators | importance | | | | | | Modification Comments |
| --- | --- | --- | --- | --- | --- | --- | --- | --- |
|  |  | 5 | 4 | 3 | 2 | 1 |  | |
| 1. Admission requirements | 1.1 Basic conditions |  |  |  |  |  |  | |
|  | 1.2 Literacy conditions |  |  |  |  |  |  | |
| Suggested Additions | |  | | | | | | |
| 2. Training objectives | 2.1 Knowledge objectives |  |  |  |  |  |  | |
|  | **2.2 Capability objectives** |  |  |  |  |  |  | |
|  | 2.3 Literacy goals |  |  |  |  |  |  | |
| Suggested Additions | |  | | | | | | |
| 3. Trainer qualifications | 3.1 Basic conditions |  |  |  |  |  |  | |
|  | 3.2 Literacy conditions |  |  |  |  |  |  | |
| Suggested Additions | |  | | | | | | |
| 4. Training method and time | 4.1 Theoretical training methods |  |  |  |  |  |  | |
|  | 4.2 Practical training methods |  |  |  |  |  |  | |
|  | 4.3 Training time |  |  |  |  |  |  | |
| Suggested Additions | |  | | | | | | |
| 5. Assessment and evaluation | 5.1 Assessment and evaluation of the training process |  |  |  |  |  |  | |
|  | 5.2 Assessment and evaluation of training completion |  |  |  |  |  |  | |
| Suggested Additions | |  | | | | | | |

**Table 1-3. Level 3 indicators of the CSSD specialized nurse training process system**

| Level 1 indicators | Level 2 indicators | Level 3 indicators | importance | | | | | | | | | Modification Comments |
| --- | --- | --- | --- | --- | --- | --- | --- | --- | --- | --- | --- | --- |
|  |  |  | 5 | | 4 | | 3 | | | 2 | 1 |  |
| 1. Access conditions | 1.1 Basic conditions | 1.1.1 College degree or above |  | |  | |  | | |  |  |  |
|  |  | 1.1.2 Have the nurse practice certificate, nurse title or above |  | |  | |  | | |  |  |  |
|  |  | **1.1.3 Working in this major for more than 2 years** |  | |  | |  | | |  |  |  |
|  |  | 1.1.4 My application and recommendation by the hospital |  | |  | |  | | |  |  |  |
|  |  | Suggested Additions |  | | | | | | | | | |
|  | 1.2 Literacy conditions | 1.2.1 Healthy, loving and identifying with dedication |  | |  | |  | | |  |  |  |
|  |  | **1.2.2 Abide by the discipline, respect the teachers, and complete the training plan on time** |  | |  | |  | | |  |  |  |
|  |  | 1.2.3Cooperation, thinking, ready to help others (New) |  | |  | |  | | |  |  |  |
|  |  | Suggested Additions |  | | | | | | | | | |
| 2. Training objectives | 2.1 Knowledge objectives | 2.1.1 Master the CSSD infection prevention and quality control |  | |  | |  | | |  |  |  |
|  |  | 2.1.2 Master the working principle of the CSSD equipment |  | |  | |  | | |  |  |  |
|  |  | 2.1.3 Master the performance and management requirements of reusable surgical instruments |  | |  | |  | | |  |  |  |
|  |  | 2.1.4 Master the sterilization monitoring and management requirements (New) |  | |  | |  | | |  |  |  |
|  |  | 2.1.5 Master the CSSD building layout and equipment and facilities (New) |  | |  | |  | | |  |  |  |
|  |  | Suggested Additions |  | | | | | | | | | |
|  | 2.2 Capability objectives | 2.2.1 Master the reprocessing process of specialized surgical instruments |  | |  | |  | | |  |  |  |
|  |  | 2.2.2 Master the daily use and maintenance of the equipment and facilities |  | |  | |  | | |  |  |  |
|  |  | 2.2.3Master CSSD quality monitoring technology (New) |  | |  | |  | | |  |  |  |
|  |  | **2.2.4 Master the principles and skills of communication** |  | |  | |  | | |  |  |  |
|  |  | **2.2.5 Ability to carry out scientific research** |  | |  | |  | | |  |  |  |
|  |  | **2.2.6 Ability to carry out teaching** |  | |  | |  | | |  |  |  |
|  |  | Suggested Additions |  | | | | | | | | | |
|  | 2.3 Literacy goals | 2.3.1 Have the spirit of prudence and treat daily work with a rigorous attitude |  | |  | |  | | |  |  |  |
|  |  | 2.3.2 Keep a positive and optimistic attitude and conduct self-debugging to meet the team development |  | |  | |  | | |  |  |  |
|  |  | 2.3.3 Pay attention to the professional development trends, and constantly improve yourself |  | |  | |  | | |  |  |  |
|  |  | 2.3.4 Compliance with ethical and legal and regulatory requirements |  | |  | |  | | |  |  |  |
|  |  | 2.3.5 has theCritical thinking skills (New) |  | |  | |  | | |  |  |  |
|  |  | Suggested Additions |  | | | | | | | | | |
| 3. Trainer qualifications | 3.1 Basic conditions | 3.1.1 Bachelor degree or above |  | |  | |  | | |  |  |  |
|  |  | 3.1.2 Intermediate or above professional title |  | |  | |  | | |  |  |  |
|  |  | 3.1.3 Obtain relevant professional qualification certificates |  | |  | |  | | |  |  |  |
|  |  | **3.1.4 The major is elimination / hospital awareness / management / teaching / scientific research / endoscopy / operating room** |  | |  | |  | | |  |  |  |
|  |  | **3.1.5 More than 5 years of relevant professional work experience** |  | |  | |  | | |  |  |  |
|  |  | **3.1.6 More than 3 years of teaching experience** |  | |  | |  | | |  |  |  |
|  |  | **3.1.7 The working unit is a grade A hospital** |  | |  | |  | | |  |  |  |
|  |  | Suggested Additions |  | | | | | | | | | |
|  | 3.2 Literacy conditions | 3.2.1 Love my major and love my teaching work |  | |  | |  | | |  |  |  |
|  |  | 3.2.2 Past students have not put forward negative opinions against medical ethics and teachers' quality |  | |  | |  | | |  |  |  |
|  |  | Suggested Additions |  | | | | | | | | | |
| 4. Training methods and duration | 4.1 Theoretical training methods | 4.1.1 Multimedia teaching |  | |  | |  | | |  |  |  |
|  |  | 4.1.2 Work visit |  | |  | |  | | |  |  |  |
|  |  | 4.1.3 Case analysis group discussion |  | |  | |  | | |  |  |  |
|  |  | 4.1.4 PBL teaching |  | |  | |  | | |  |  |  |
|  |  | 4.1.5 Flipped classroom |  | |  | |  | | |  |  |  |
|  |  | Suggested Additions |  | | | | | | | | | |
|  | 4.2 Practical training methods | 4.2.1 Clinical one-site teaching |  | |  | |  | | |  |  |  |
|  |  | 4.2.2 Scenario simulation teaching |  | |  | |  | | |  |  |  |
|  |  | 4.2.3 Operation instruction |  | |  | |  | | |  |  |  |
|  |  | 4.2.4 Joint teaching of multiple departments (related departments: such as operating room, stomatology, endoscopy) (New) |  | |  | |  | | |  |  |  |
|  |  | Suggested Additions |  | | | | | | | | | |
|  | 4.3 Training duration | 4.3.1 The theoretical training lasts one month and the total duration is 160 hours |  | |  | |  | | |  |  |  |
|  |  | **4.3.2 The practical training lasts for 2 months with a total of 320 hours** |  | |  | |  | | |  |  |  |
|  |  | Suggested Additions |  | | | | | | | | | |
| 5. Assessment and evaluation | 5.1 Assessment and evaluation of the training process | 5.1.1 Medical ethics |  | |  | |  | | |  |  |  |
|  |  | 5.1.2 Professional Literacy |  | |  | |  | | |  |  |  |
|  |  | 5.1.3 Daily performance (attendance) |  |  | |  | |  |  | | |  |
|  |  | **5.1.4 Completion of phased goals** |  |  | |  | |  |  | | |  |
|  |  | Suggested Additions |  | | | | | | | | | |
|  | 5.2 Assessment and evaluation of training completion | 5.2.1 Assessment of theoretical knowledge |  |  | |  | |  |  | | |  |
|  |  | 5.2.2 Assessment of practical skills |  |  | |  | |  |  | | |  |
|  |  | 5.2.3 Small lectures |  |  | |  | |  |  | | |  |
|  |  | Suggested Additions |  | | | | | | | | | |

**Part 2: Construction of the Training Content Indicator System for Specialized Nurses in the Central Sterile Supply Department**

Table 2-1 . Level 1 indicators of the CSSD specialized nurse training content indicator system

| Level 1 indicators | importance | | | | | Modification Comments |
| --- | --- | --- | --- | --- | --- | --- |
|  | 5 | 4 | 3 | 2 | 1 |  |
| 1. knowledge |  |  |  |  |  |  |
| **2. Technology** |  |  |  |  |  |  |
| 3. Ability |  |  |  |  |  |  |
| 4. Feature |  |  |  |  |  |  |
| Suggested Additions |  | | | | | |

Table 2-2. Leve 2 indicators of the CSSD specialized nurse training content indicator system

| Level 1 indicators | Level 2 indicators | importance | | | | | Modification Comments |
| --- | --- | --- | --- | --- | --- | --- | --- |
|  |  | 5 | 4 | 3 | 2 | 1 |  |
| 1. knowledge | 1.1 Overview of the disinfection supply center |  |  |  |  |  |  |
|  | 1.2 Basic knowledge related to disinfection supply |  |  |  |  |  |  |
|  | 1.3 Knowledge of disinfection and sterilization |  |  |  |  |  |  |
|  | 1.4 Hospital infection and occupational protection (New) |  |  |  |  |  |  |
| Suggested Additions | |  | | | | | |
| 2. Technology | 2.1 Preprocessing technology |  |  |  |  |  |  |
|  | 2.2 Device recovery technology |  |  |  |  |  |  |
|  | 2.3 Device classification technique |  |  |  |  |  |  |
|  | 2.4 Device cleaning and disinfection technology |  |  |  |  |  |  |
|  | 2.5 Device Drying technique |  |  |  |  |  |  |
|  | 2.6 Device inspection and maintenance technology |  |  |  |  |  |  |
|  | 2.7 Device packaging technology |  |  |  |  |  |  |
|  | 2.8 Device Sterilization technique |  |  |  |  |  |  |
|  | 2.9 Device storage and distribution |  |  |  |  |  |  |
|  | 2.10 Logistics and distribution of devices |  |  |  |  |  |  |
|  | 2.11 Quality monitoring technology |  |  |  |  |  |  |
|  | 2.12 Information processing technology (New) |  |  |  |  |  |  |
| Suggested Additions | |  | | | | | |
| 3. Ability | 3.1 Management ability |  |  |  |  |  |  |
|  | 3.2 Risk management and control ability |  |  |  |  |  |  |
|  | 3.3 Learning ability |  |  |  |  |  |  |
|  | 3.4 Scientific research ability |  |  |  |  |  |  |
|  | 3.5 Teaching ability |  |  |  |  |  |  |
|  | 3.6 Communication skills |  |  |  |  |  |  |
| Suggested Additions | |  | | | | | |
| 4. Feature | 4.1 Code of professional ethics |  |  |  |  |  |  |
|  | 4.2 Personal literacy |  |  |  |  |  |  |
| Suggested Additions | |  | | | | | |

Table 2-3. Leve 3indicators of the CSSD specialized nurse training content indicator system

| Level 1 indicators | Level 2 indicators | Level 3 indicators | importance | | | | | Modification Comments |
| --- | --- | --- | --- | --- | --- | --- | --- | --- |
|  |  |  | 5 | 4 | 3 | 2 | 1 |  |
| 1. knowledge | 1.1 Overview of the disinfection supply center | 1.1.1 Development course of CSSD at home and abroad |  |  |  |  |  |  |
|  |  | 1.1.2 Management mode of CSSD |  |  |  |  |  |  |
|  |  | 1.1.3 Building and layout requirements of CSSD |  |  |  |  |  |  |
|  |  | 1.1.4 Job responsibilities and system of CSSD |  |  |  |  |  |  |
|  |  | 1.1.5 Relevant laws and regulations and industry standards of CSSD |  |  |  |  |  |  |
|  | Suggested Additions | |  | | | | | |
|  | 1.2 Basic knowledge related to disinfection supply | 1.2.1 Basic knowledge of medical devices |  |  |  |  |  |  |
|  |  | 1.2.2 Basic principles of CSSD equipment and facilities |  |  |  |  |  |  |
|  |  | 1.2.3Knowledge of disinfection supply processing process (New) |  |  |  |  |  |  |
|  | Suggested Additions | |  | | | | | |
|  | 1.3 Knowledge of disinfection and sterilization | 1.3.1 Common microbial species |  |  |  |  |  |  |
|  |  | 1.3.2 Common methods for disinfection and sterilization |  |  |  |  |  |  |
|  | Suggested Additions | |  | | | | | |
|  | 1.4 Hospital infection and occupational protection | 1.4.1Hospital infection control (New) |  |  |  |  |  |  |
|  |  | 1.4.2 Prevention of infection and occupational protection in CSSD |  |  |  |  |  |  |
|  |  | 1.4.3Management of the occupational exposure |  |  |  |  |  |  |
|  | Suggested Additions | |  | | | | | |
| 2. Skills | 2.1 Preprocessing technology | 2.1.1 Treatment on site |  |  |  |  |  |  |
|  |  | 2.1.2 Pretreatment before cleaning |  |  |  |  |  |  |
|  | Suggested Additions | |  | | | | | |
|  | 2.2 Device recovery technology | 2.2.1 Recovery of conventional devices |  |  |  |  |  |  |
|  |  | 2.2.2 Recovery of specialized precision equipment |  |  |  |  |  |  |
|  |  | 2.2.3 Recovery of special contaminated devices |  |  |  |  |  |  |
|  |  | 2.2.4 Recovery of implants and external medical devices |  |  |  |  |  |  |
|  |  | 2.2.5 Recovery of soft endoscopic and hard endoscopic instruments |  |  |  |  |  |  |
|  |  | 2.2.6 Recovery of cross-hospital devices |  |  |  |  |  |  |
|  |  | **2.2.7 Disposal of recovery and transport tools** |  |  |  |  |  |  |
|  |  | 2.2.8 Application of Electronic Information Traceability System in Recovery Process (New) |  |  |  |  |  |  |
|  | Suggested Additions | |  | | | | | |
|  | 2.3 Device classification technique | 2.3.1 Classification according to the device material |  |  |  |  |  |  |
|  |  | 2.3.2 Classification according to the device structure |  |  |  |  |  |  |
|  |  | 2.3.3 Classification according to the degree of device contamination |  |  |  |  |  |  |
|  | Suggested Additions | |  | | | | | |
|  | 2.4 Device cleaning and disinfection technology | 2.4.1 Selection of cleaning and disinfection methods (New) |  |  |  |  |  |  |
|  |  | 2.4.2 Manual cleaning techniques (including cleaning of soft endoscope, robotic surgical instruments, endoscopic instruments, etc.) |  |  |  |  |  |  |
|  |  | 2.4.3 Mechanical cleaning technology |  |  |  |  |  |  |
|  |  | 2.4.4 Selection of cleaning agent and disinfectant |  |  |  |  |  |  |
|  |  | 2.4.5 Configuration of cleaning agent and disinfectant |  |  |  |  |  |  |
|  |  | 2.4.6 Cleaning and disinfection process of special infected devices |  |  |  |  |  |  |
|  |  | 2.4.7 Use and daily maintenance of ultrasonic cleaning machine |  |  |  |  |  |  |
|  |  | 2.4.8 Use and daily maintenance of mechanical cleaning machine |  |  |  |  |  |  |
|  |  | 2.4.9 Use and daily maintenance of water treatment equipment |  |  |  |  |  |  |
|  |  | 2.4.10 Use and daily maintenance of other cleaning and disinfection techniques / equipment (e. g. acidified water,Steam cleaning machine)(New) |  |  |  |  |  |  |
|  |  | 2.4.11 Application of Electronic Information traceability system in cleaning and disinfection (New) |  |  |  |  |  |  |
|  | Suggested Additions | |  | | | | | |
|  | 2.5 Device Drying technique | 2.5.1 Selection of drying mode |  |  |  |  |  |  |
|  |  | 2.5.2 Judgment of the drying effect |  |  |  |  |  |  |
|  |  | 2.5.3 Use and daily maintenance of drying equipment |  |  |  |  |  |  |
|  | Suggested Additions | |  | | | | | |
|  | 2.6 Device inspection and maintenance | 2.6.1 Inspection of the device cleanliness |  |  |  |  |  |  |
|  |  | 2.6.2 Check of the functional status of the device |  |  |  |  |  |  |
|  |  | 2.6.3 Insulation performance test of the device |  |  |  |  |  |  |
|  |  | 2.6.4 Disassembly and assembly of the device |  |  |  |  |  |  |
|  |  | 2.6.5 Maintenance technology of the devices |  |  |  |  |  |  |
|  | Suggested Additions | |  | | | | | |
|  | 2.7 Device packaging technology | 2.7.1 Selection of packaging materials |  |  |  |  |  |  |
|  |  | 2.7.2 Selection and application of packaging method |  |  |  |  |  |  |
|  |  | 2.7.3 Use and daily maintenance of medical sealing machine |  |  |  |  |  |  |
|  |  | 2.7.4 Setting of packaging identification of sterilized items |  |  |  |  |  |  |
|  |  | 2.7.5 Inspection of packaging quality |  |  |  |  |  |  |
|  |  | 2.7.6 Application of Electronic Information Traceability System in Packaging Process ( New) |  |  |  |  |  |  |
|  | Suggested Additions | |  | | | | | |
|  | 2.8 Device Sterilization technique | 2.8.1 The loading and unloading of sterilized articles |  |  |  |  |  |  |
|  |  | 2.8.2 Use and daily maintenance of pressure steam sterilizer |  |  |  |  |  |  |
|  |  | 2.8.3 Use and daily maintenance of ethylene oxide sterilizer |  |  |  |  |  |  |
|  |  | 2.8.4 Use and daily maintenance of hydrogen peroxide low-temperature plasma sterilizer |  |  |  |  |  |  |
|  |  | 2.8.5 Use and daily maintenance of low-temperature steam formaldehyde sterilizer |  |  |  |  |  |  |
|  |  | 2.8.6 Interpretation of sterilization results (physical, chemical, biological) |  |  |  |  |  |  |
|  |  | 2.8.7 SterilizationPeriodic maintenance and performance monitoring of the equipment (New) |  |  |  |  |  |  |
|  |  | 2.8.8 Application of Electronic Information Tracing System for sterilization process (New) |  |  |  |  |  |  |
|  | Suggested Additions | |  | | | | | |
|  | 2.9 Device storage and distribution | 2.9.1 Storage and validity period of sterile items |  |  |  |  |  |  |
|  |  | 2.9.2 Distribution process of sterile articles |  |  |  |  |  |  |
|  |  | 2.9.3 Distribution process of emergency delivery devices |  |  |  |  |  |  |
|  |  | 2.9.4 Application of Electronic Information traceability system in the distribution process (New) |  |  |  |  |  |  |
|  | Suggested Additions | |  | | | | | |
|  | 2.10 Logistics and distribution of devices | 2.10.1 Device in-hospital transfer |  |  |  |  |  |  |
|  |  | 2.10.2 Hospital-level device transfer |  |  |  |  |  |  |
|  | Suggested Additions | |  | | | | | |
|  | 2.11 Quality monitoring technology | 2.11.1 Water quality monitoring |  |  |  |  |  |  |
|  |  | 2.11.2Steam quality monitoring (New) |  |  |  |  |  |  |
|  |  | 2.11.3 Monitoring of the environment and staff hand hygiene in CSSD |  |  |  |  |  |  |
|  |  | 2.11.4 Monitoring technology of cleaning quality and interpretation of results |  |  |  |  |  |  |
|  |  | 2.11.5 Disinfection quality monitoring technology (New) |  |  |  |  |  |  |
|  |  | 2.11.6 Monitoring technology of sterilization quality |  |  |  |  |  |  |
|  |  | 2.11.7 Recall and disposal process of unqualified items |  |  |  |  |  |  |
|  | Suggested Additions | |  | | | | | |
| 3. Ability | 3.1 Management ability | 3.1.1 CSSD material management |  |  |  |  |  |  |
|  |  | 3.1.2 CSSD organization and management |  |  |  |  |  |  |
|  |  | 3.1.3 Performance management of CSSD |  |  |  |  |  |  |
|  |  | 3.1.4 CSSD Cost management |  |  |  |  |  |  |
|  |  | 3.1.5 CSSD equipment and facilities management |  |  |  |  |  |  |
|  |  | 3.1.6 Adverse event management of CSSD |  |  |  |  |  |  |
|  |  | 3.1.7 CSSD information construction and management |  |  |  |  |  |  |
|  |  | 3.1.8 Management of relevant documents and records |  |  |  |  |  |  |
|  |  | 3.1.9 Use of common quality management tools |  |  |  |  |  |  |
|  | Suggested Additions | |  | | | | | |
|  | 3.2 Risk management and control ability | 3.2.1 Risk prevention capability |  |  |  |  |  |  |
|  |  | 3.2.2 Emergency control capability |  |  |  |  |  |  |
|  | Suggested Additions | |  | | | | | |
|  | 3.3 Learning ability | 3.3.1 Learning and application of new technology and new business |  |  |  |  |  |  |
|  |  | 3.3.2 Ability to obtain professional information |  |  |  |  |  |  |
|  | Suggested Additions | |  | | | | | |
|  | 3.4 Scientific research ability | 3.4.1 Literature retrieval and reading ability |  |  |  |  |  |  |
|  |  | 3.4.2 Scientific research topics |  |  |  |  |  |  |
|  |  | 3.4.3 Methods of scientific research and design |  |  |  |  |  |  |
|  |  | 3.4.4 Collection and management of scientific research data |  |  |  |  |  |  |
|  |  | 3.4.5 Common statistical methods |  |  |  |  |  |  |
|  |  | 3.4.6 Writing of the nursing paper |  |  |  |  |  |  |
|  |  | 3.4.7 New technologies and new business development |  |  |  |  |  |  |
|  |  | 3.4.8 Project development and management |  |  |  |  |  |  |
|  | Suggested Additions | |  | | | | | |
|  | 3.5 Teaching ability | 3.5.1 Organization and management of clinical teaching |  |  |  |  |  |  |
|  |  | 3.5.2 Clinical teaching methods |  |  |  |  |  |  |
|  |  | 3.5.3 Clinical teaching skills |  |  |  |  |  |  |
|  |  | 3.5.4 Evaluation of clinical teaching |  |  |  |  |  |  |
|  | Suggested Additions | |  | | | | | |
|  | 3.6 Communication skills | 3.6.1 Communication ability with the service department |  |  |  |  |  |  |
|  |  | 3.6.2 Communication ability with foreign consumer consumption service units |  |  |  |  |  |  |
|  |  | 3.6.3 Communication ability with the guarantee department |  |  |  |  |  |  |
|  |  | 3.6.4 Communication skills with the personnel in the department |  |  |  |  |  |  |
|  | Suggested Additions | |  | | | | | |
| 4. Feature | 4.1 Code of professional ethics | 4.1.1 Spirit of prudence |  |  |  |  |  |  |
|  |  | 4.1.2 Prevention of job burnout |  |  |  |  |  |  |
|  |  | 4.1.3 Empathy and responsibility |  |  |  |  |  |  |
|  | Suggested Additions | |  | | | | | |
|  | 4.2 Personal literacy | 4.2.1 Emotional management ability |  |  |  |  |  |  |
|  |  | 4.2.2 Suitability |  |  |  |  |  |  |
|  |  | 4.2.3 Decision-making ability |  |  |  |  |  |  |
|  | Suggested Additions | |  | | | | | |

**Expert name:**
